# Protective Role of Myeloid Cells Expressing a G-CSF Receptor Polymorphism in an Induced Model of Lupus

**DOI:** 10.3389/fimmu.2018.01053

**Published:** 2018-05-09

**Authors:** Ramya Sivakumar, Georges Abboud, Clayton E. Mathews, Mark A. Atkinson, Laurence Morel

**Affiliations:** Department of Pathology, Immunology, Laboratory Medicine, University of Florida Diabetes Institute, University of Florida, Gainesville, FL, United States

**Keywords:** G-CSF-R, lupus, neutrophils, tolerogenic dendritic cells, suppressive allele

## Abstract

The genetic analysis of the lupus-prone NZM2410 mouse has identified a suppressor locus, *Sle2c2*, which confers resistance to spontaneous lupus in combination with NZM2410 susceptibility loci, or in the chronic graft-versus-host disease (cGVHD) induced model of lupus in the B6.*Sle2c2* congenic strain. The candidate gene for *Sle2c2*, the *Csf3r* gene encoding the granulocyte colony-stimulating factor receptor (G-CSF-R/CD114), was validated when cGVHD was restored in B6.*Sle2c2* mice after treatment with G-CSF. The goal of the project reported herein was to investigate the myeloid cells that confer resistance to cGVHD and to ascertain if the mechanism behind their suppression involves the G-CSF pathway. We showed that despite expressing the highest levels of G-CSF-R, neutrophils play only a modest role in the autoimmune activation induced by cGVHD. We also found reduced expression levels of G-CSF-R on the surface of dendritic cells (DCs) and a differential distribution of DC subsets in response to cGVHD in B6.*Sle2c2* versus B6 mice. The CD8α^+^ DC subset, known for its tolerogenic phenotype, was expanded upon induction of cGVHD in B6.*Sle2c2* mice. In addition, the deficiency of CD8α^+^ DC subset enhanced the severity of cGVHD in B6.*Batf3*^−/−^ and B6*.Sle2c2* mice, confirming their role in suppression of cGVHD. B6.*Sle2c2*DCs presented lowered activation and antigen presentation abilities and expressed lower levels of genes associated with DC activation and maturation. Exposure to exogenous G-CSF reversed the majority of these phenotypes, suggesting that tolerogenic DCs maintained through a defective G-CSF-R pathway mediated the resistance to cGVHD in B6.*Sle2c2* mice.

## Introduction

Systemic lupus erythematosus (SLE) is a chronic autoimmune disorder with a complex etiology. It is commonly accepted that SLE results from a combination of genetic and environmental factors. Almost all key immunological pathways and immune cells have been implicated in lupus (1, 2). Defects ranging from improper clearance of apoptotic debris to the aberrant activation of both innate and adaptive immune cells, as well as genetic polymorphisms in these pathways have been linked with the pathogenesis of lupus (1–4). Most relevant to this study, multiple defects have been found in the activation and subset distribution of dendritic cells (DCs) in both lupus mouse models and patients (5).

Genome-wide association studies have identified nearly 80 polymorphic loci that are associated with lupus (6). Mouse models have largely contributed to the understanding of the genetic complexity in lupus and to the identification of functional pathways and putative targets for therapy (7, 8). A linkage analysis performed in the NZM2410 model identified three susceptibility loci (*Sle1*–*Sle3*) as well as one protective locus (*Sle4* or *Sles1*) linked to lupus nephritis (9). Phenotypes and susceptibility genes associated with these loci have helped in identifying novel pathways leading to systemic autoimmunity (7). The characterization of suppressive loci has also the potential to unravel disease etiology, which integrates the combination of susceptibility and resistance alleles. Indeed, the suppressive NZW-derived *Sle4/Sles1* locus located in the MHC class II locus (10–12) is likely to correspond to the HLA class II locus identified in lupus patients (13).

This study focuses on the NZM2410/NZB-derived suppressor locus *Sle2c2*, which is located at the telomeric end of *Sle2* and confers resistance to spontaneous lupus (14) as well as to a chronic graft-versus-host disease (cGVHD) induced model of SLE (15). cGVHD is suppressed in B6.*Sle2c2* mice through non-B non-T hematopoietic cells, and a non-synonymous polymorphism in the *Csf3r* gene encoding for the granulocyte colony-stimulating factor receptor (G-CSF-R/CD114) was identified as the top candidate gene responsible for disease suppression (15). The rs13477964 polymorphism converts serine to asparagine (S379N) in the fibronectin 3 domain located in the extracellular portion of G-CSF-R. This variation has the ability to affect the stability or orientation of the receptor dimer during ligand binding (16). Rescue of cGVHD by exogenous G-CSF validated the involvement of the G-CSF-R pathway in the B6.*Sle2c2* mice and suggested that protection was mediated by a loss of function allele (17).

The expression of G-CSF-R is highest on neutrophils (PMNs) and myeloid progenitor cells, but G-CSF-R is also found on monocytes, DCs, and activated lymphocytes (18, 19). The immunological functions of the G-CSF/G-CSFR pathway are complex (20). G-CSF is well known for its anti-inflammatory effect on T cells, monocytes, and DCs (21–24) and for its immunoregulatory role in type 1 diabetes (25–27) and multiple sclerosis (28, 29). In lupus, however, chronic low doses of G-CSF have accelerated disease while a high dose of G-CSF prevented nephritis in the MRL/lpr model (30). Furthermore, when administered to neutropenic SLE patients, G-CSF induced flares (31, 32). G-CSF treatment also resulted in dual outcomes in experimental models of acute graft-versus-host disease (aGVHD): pretreatment of donor mice with G-CSF reduced aGVHD severity (33). However, G-CSF administered after total body irradiation of recipient mice exacerbated aGVHD disease outcomes due to an increased expression of G-CSF-R on antigen presenting cells (34).

All the myeloid cell subsets that express G-CSF-R on their surface, including DCs, monocytes, macrophages (Mϕ), and neutrophils, have been implicated in the pathogenesis of lupus (35–39). We hypothesized that the defective response of myeloid cells to endogenous G-CSF was responsible for suppressing cGVHD in B6.*Sle2c2* mice. We determined that the depletion of neutrophils had a minimal effect in cGVHD pathogenesis, which focused the study toward DCs. Conventional DCs (cDCs) are divided into CD8α^+^ DCs and CD11b^+^ DCs, which are further categorized into CD4^+^ and CD8α^−^CD4^−^ (DN) DC subsets (40). CD8α^+^ DCs cross-present antigens and activate cytotoxic CD8^+^ T cells (41). CD11b^+^ DN DCs are generally associated with priming CD4^+^ T cells (42). cGVHD suppression correlated with an increased frequency of CD8α^+^ DCs and a decreased frequency of DN DCs in the spleen of B6.*Sle2c2* mice. *Sle2c2* DCs expressed an anti-inflammatory gene signature and decreased CD4^+^ T cell activation with a preferential skewing toward regulatory phenotypes. The protective phenotype of *Sle2c2* DCs was reversed by G-CSF, confirming that B6.*Sle2c2* mice carry a loss of function allele of *Csf3r*. Finally, the protective role of CD8α^+^ DCs in cGVHD was demonstrated both in B6.*Batf3*^−/−^ and B6.*Sle2c2* mice, further confirming the tolerogenic role of this DC subset. Overall, these results show that the expression of the *Sle2c2 Csf3r* allele confers autoimmune suppression through the expansion of tolerogenic DCs.

## Materials and Methods

### Mice

C57BL/6J (B6) mice, B6.C-H2-Ab1bm12/KhEgJ (bm12), B6.Cg-Tg (TcraTcrb)425Cbn/J(OT-II), and B6.129S-*Batf3*tm1Kmm/J (B6.*Batf3*^−/−^) mice were obtained from the Jackson Laboratories. B6.*Sle2c2* mice have been previously described (14). Both males and females were used between 2 and 6 months of age. Gender and age were matched between strains for each experiment. All mice were bred and maintained at the University of Florida in specific pathogen-free conditions. This study was carried out in strict accordance with the recommendations in the Guide for the Care and Use of Laboratory Animals of the Animal Welfare Act and the National Institutes of Health guidelines for the care and use of animals in biomedical research. All animal protocols were approved by the Institutional Animal Care and Use Committee of the University of Florida, Gainesville (OLAW Assurance # A3377-01).

### cGVHD Induction and Analysis

Chronic graft-versus-host disease was induced according to an established protocol (43). Briefly, 50–80 × 10^6^ bm12 splenocytes were transferred intraperitoneally into B6, B6.*Sle2c2*, or *Batf3*^−^*^/^*^−^ mice. Serum was collected weekly up to 3 weeks after induction. Groups of at least five mice were sacrificed 7, 14, and 21 days after transfer to assess the phenotype of the splenocytes from recipient mice as previously described (15, 17). In some experiments, the cGVHD-induced B6 and B6*.Sle2c2* mice were treated subcutaneously with 12 µg of pegylated human G-CSF (Neulasta^®^, huG-CSF, Amgen, Thousand Oaks, CA, USA), referred to as huG-CSF, diluted in 5% dextrose, or 5% dextrose alone as control, twice a week starting on the day of induction. To deplete PMNs, mice were treated as described (44) with 1A8 0.5 mg of anti-Ly6G antibody (Bio X Cell) or rat IgG2b isotype control thrice weekly starting on the day of induction.

### CD8α^+^ DC Depletion or Transfer During cGVHD

During cGVHD induction some B6.*Sle2c2* mice were depleted of spleen CD8α^+^DCs using anti-CD8α depleting antibody (clone 2.43, 300 µg/mouse) thrice weekly starting from day 3 and until day 17. This method caused 50% depletion of CD8α^+^ DCs. Alternatively, B6.*Batf3*^−/−^ mice received *via* i.v. route 50–70 × 10^3^ CD8α^+^ DCs (>92% purity) sorted from a pool of three naïve B6.*Sle2c2* mice on days 2, 7, and 14 post-cGVHD induction.

### Flow Cytometry

Spleens were digested with collagenase (Roche) or Liberase (Sigma) in NaHCO3-free RPMI1640 (Gibco). Single cell suspensions treated with 155 mM NH4Cl to lyse red blood cells were passed through a pre-separation filter (Miltenyi Biotec) to remove debris. Fc receptors were blocked with 10% rabbit serum and anti-CD16/32 (2.4G2) in staining buffer (2.5% FBS, 0.05% sodium azide in PBS). Cells were then stained for 30 min on ice with predetermined amounts of the following fluorophore-conjugated or biotinylated antibodies purchased from BD biosciences, Biolegend, eBiosciences, Life Technologies or Santa Cruz Biotechnology: B220 (RA3-6B2), CD4 (RM4-5), CD8α (53-6.7), CD11b (M1/70), CD11c (HL3), CD25 (7D4), CD40 (HM40-3), CD44 (IM7), CD45 (30-F11), CD62L (MEL-14), CD69 (H1.2F3), CD80 (16-10A1); CD86 (GL1), CD127 (SB/199), CD154 (MR1), CXCR3 (CXCR3-173), FOXP3 (FJK-16s), F4-80 (BM8), ICOS (C398.4A), Ki-67 (16A8), KLRG-1 (MAFA), Ly6G (1A8), MHC-II (M5/114.15.2), and MHC-II I-Ab (AFG 1201). Biotinylated antibodies were detected with streptavidin conjugated to phycoerythrin-Cy7 or allophycocyanin (APC)-Cy7. For detection of G-CSF-R, a polyclonal goat antibody (sc-323898) was used followed by donkey anti-goat IgG FITC. Data were acquired using either BD LSR Fortessa (BD biosciences) or CyAN ADP (Beckman Coulter), and analyzed with Flowjo (Treestar). At least 10,000 events were acquired per sample from *in vitro* assays, and a minimum of 100,000 events were acquired per *ex vivo* sample. Dead cells and doublets were gated out using live-dead dye and/or scatter characteristics. DC subsets were sorted on a FACS-Aria cell sorter (BD biosciences) with the antibody panels indicated in the text.

### Generation of Bone Marrow-Derived DCs (BMDCs)

Briefly, bone marrow single cell suspensions were cultured with 10 ng/mL of GM-CSF and IL-4 (R&D systems) for 6 days. One half of the media was replenished on day 3 and cells were harvested on day 6. BMDCs were also prepared from B6 and B6.*Sle2c2* mice on day 4 after treatment with a single dose of 12 µg of huG-CSF [Neulasta (Amgen)].

### Assessment of DC Functions

B6 and B6.*Sle2c2* mice were immunized with 100 µg NP-OVA (Biosearch Technology) in alum (1:1) 20 days after cGVHD induction. Splenic CD4^+^, CD8α^+^ and DN DCs were FACS-sorted 1 day later from three pooled mice per strain and cocultured with purified CD4^+^ T cells (Miltenyi Biotec) from OT-II mice at 1:5 ratio for 72 h, with or without of 1 µg of OVA 323–339 peptide. Similarly, CD11c^+^ BMDCs were stimulated with 0.1–1 µg/mL LPS for 24 h, or incubated with purified OT-II T cells for 24–72 h in the presence of 0.1–1 µg of OVA peptide. The phenotypes of activated DCs and cocultured T cells in these assays were determined by flow cytometry and by cytokine ELISAs.

### ELISA

Serum autoantibody levels were determined as previously described (45). Briefly, anti-dsDNA or anti-chromatin IgG were measured in 1:100 diluted sera in duplicates in plates coated with 50 µg/mL dsDNA (Sigma), followed by 10 µg/mL of total histone (Roche) for chromatin specificities. The secondary antibody was goat anti-mouse IgG conjugated to alkaline phosphatase (Southern Biotech). IL-6, IL-10, IL-12, TNF-α, and IFN-γ were measured in the supernatants collected from 24 h cultures of BMDCs stimulated with LPS with single-analyte kits (R&D systems). The supernatants from 72 h cocultures of CD11c^+^ BMDCs with OT-II T cells were assayed with the Mouse Th1/Th2/Th17 cytokines Multi-Analyte ELISArray Kit (Qiagen).

### Gene Expression

Gene expression was compared between B6 and B6.*Sle2c2* BMDCs with a PCR array containing 84 genes associated with DC activation and maturation (Qiagen PAMM-406ZA). Briefly, RNA was extracted from CD11c^+^ BMDCs using the RNeasy kit followed by the MinElute cleanup kit (Qiagen). cDNA was prepared using RT2 first strand kit reagents (Qiagen). Data were analyzed with non-supervised hierarchical clustering of the entire dataset, and the significant differences between strains were shown as a heatmap.

### Statistical Analysis

Analyses were performed using GraphPad Prism 6.0. Unless indicated, graphs show means and SDs of the mean. Comparisons were performed using two-tailed unpaired student *t* tests and two-way ANOVA. Statistical significance was defined as *p* < 0.05.

## Results

### Resistance to cGVHD in B6.*Sle2c2* Mice is Associated With an Expansion of CD8α^+^ DCs

**Figure 1:**
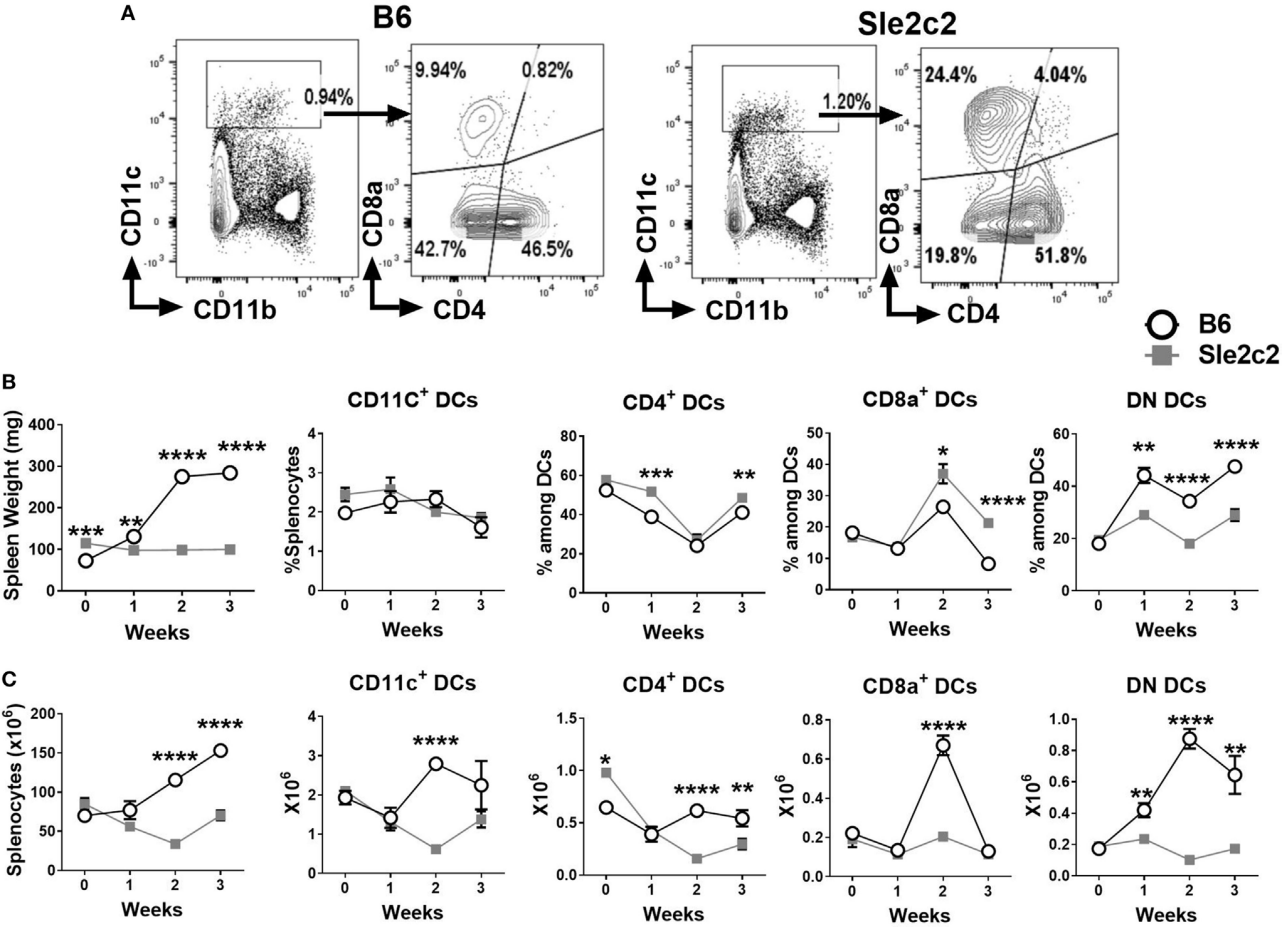
Splenic dendritic cell (DC) distribution during the course of chronic graft-versus-host disease (cGVHD) in B6 and B6.*Sle2c2* mice. **(A)** Gating strategy used to identify CD4^+^, CD8α^+^, and DN DCs from CD11c^+^ gated cells. **(B)** Spleen weight and frequency of the gated populations at the indicated time points after cGVHD induction. **(C)** Splenocytes numbers and absolute numbers of total gated populations (*N* = 5–12 per time point, *p < 0.05; **p < 0.01, ***p < 0.001, ****p < 0.0001).

Changes in myeloid cells during the course of cGVHD were assessed prior to and at 7, 14, and 21 days after induction using cohorts of B6.*Sle2c2* and B6 mice. The gating strategy used for CD4^+^, CD8α^+^ and DN DCs is shown in Figure 1A. Results were plotted in parallel with changes observed in spleen weight and splenocytes counts as cGVHD markers to correlate the myeloid cell progression with the cGVHD response (Figures 1B,C). Strain-specific differences were observed after induction of cGVHD for all DC subsets. While the total frequency of total CD11c^high^ DCs remained similar between B6 and B6.*Sle2c2* mice, there was a small increase in the frequency of CD4^+^ DCs at weeks 1 and 3 after induction, and a greater increase at week 2 and 3 for CD8α^+^ DCs in B6.*Sle2c2* mice. To the contrary, the frequency of DN DCs decreased in B6.*Sle2c2* mice throughout the 3-week course of cGVHD (Figure 1B). B6.*Sle2c2* mice presented a higher absolute number of CD4^+^ DCs compared to B6 control mice before induction, which decreased significantly during cGVHD, as opposed to B6 mice in which the population stayed relatively constant except for a dip during week 1 (Figure 1C). The numbers of the CD8α^+^ and DN DCs were similar between the two strains before induction. As a consequence of the expansion of the total number of splenocytes in B6 mice during cGVHD (Figure 1C), the number of DCs from each of the three DC subsets including total DCs was higher in B6 than B6.*Sle2c2* mice (Figure 1C). However, the number of CD8α^+^ DCs sharply decreased only in B6 mice at week 3. Overall, differences in the distribution and dynamics of DC subsets were observed between B6.*Sle2c2* and B6 mice during cGVHD autoimmune stimulation.

**Figure 2:**
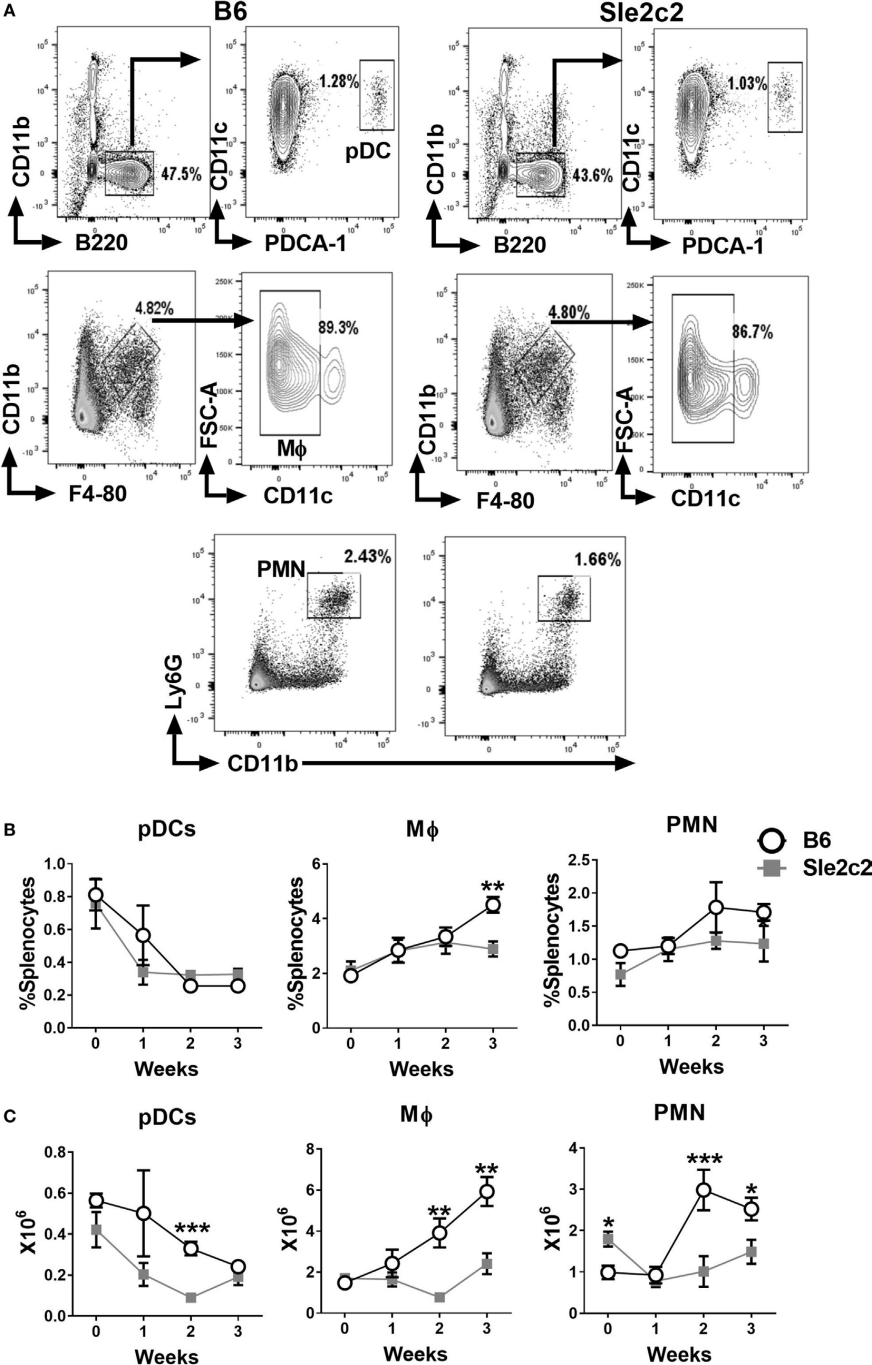
Splenic plasmacytoid dendritic cell (pDC), macrophages (Mϕ), and polymorphonuclear neutrophil (PMN) distribution during the course of chronic graft-versus-host disease (cGVHD) in B6 and B6.*Sle2c2* mice. **(A)** Gating strategy used to identify pDCs (top), Mϕ (middle), and PMN (bottom panel) from live cells. Frequency **(B)** and numbers **(C)** of the gated populations at the indicated time points after cGVHD induction (*N* = 5–12 per time point, *p < 0.05; **p < 0.01, ***p < 0.001).

The gating strategy used for CD11b^−^ B220^+^ CD11c^lo^ PDCA-1^+^ plasmacytoid DCs (pDCs), CD11b^+^ F4-80^+^ CD11c^−^ Mϕ, and Ly6G^+^ CD11b^+^ PMNs is shown in Figure 2A. The frequency of pDCs decreased after cGVHD induction in a similar manner in both strains (Figure 2B). A small increase in the frequency of B6 Mϕ was observed at week 3 (Figure 2B). The frequency of PMNs increased in a similar manner between the two strains (Figure 2B) and but the number of PMNs was higher in B6.*Sle2c2* than in B6 mice at steady state (Figure 2C). Overall, cGVHD suppression in B6.*Sle2c2* mice was associated with a higher frequency of CD8α^+^ DCs, and to a lesser extent, CD4^+^ DCs, as well as the failure to expand DN DCs as compared to B6 mice.

### B6.*Sle2c2* PMNs and DCs Express Lower Levels of G-CSF-R

**Figure 3:**
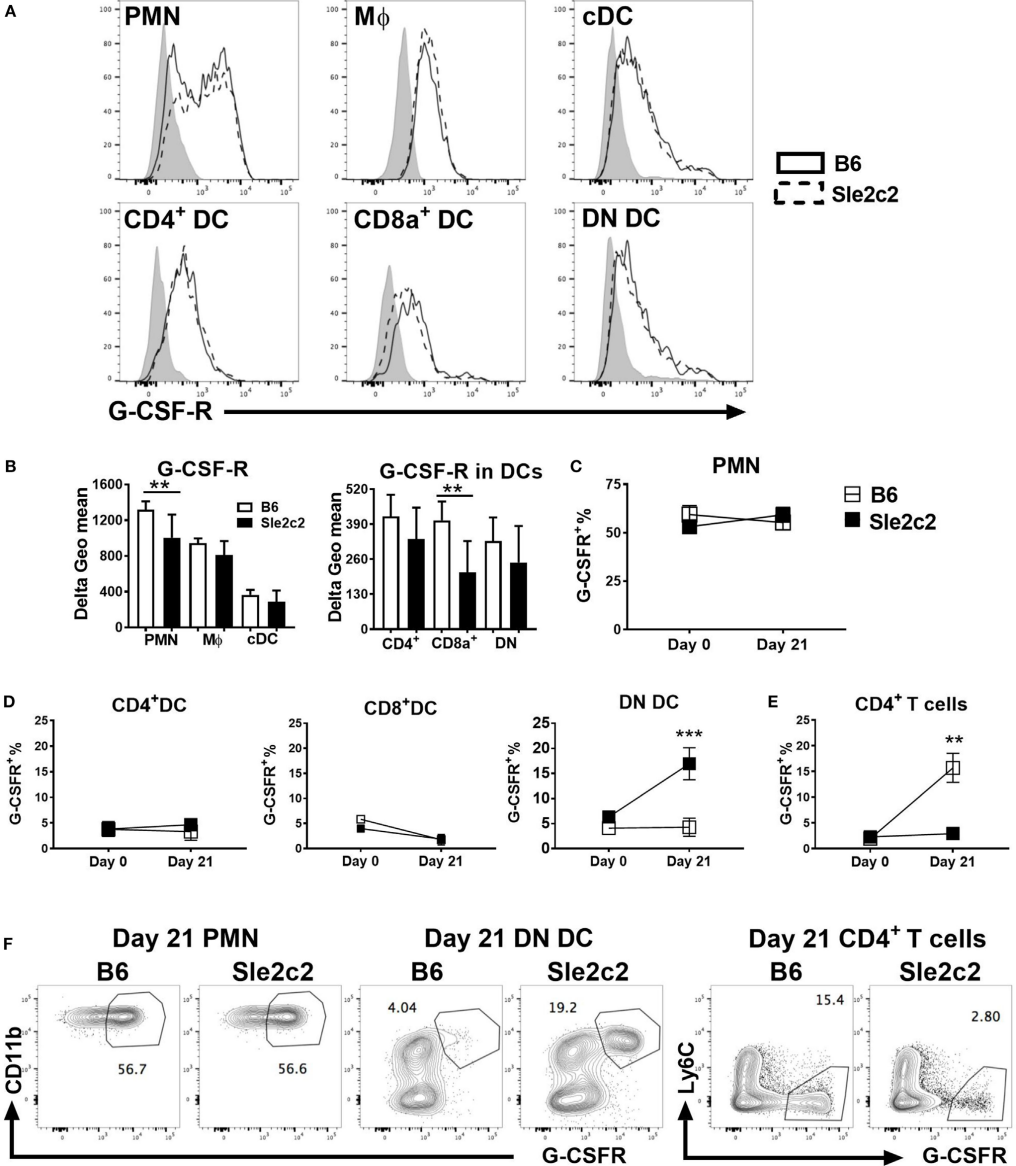
B6.*Sle2c2* mice express lower levels of G-CSF-R on polymorphonuclear neutrophils (PMNs) and dendritic cells (DCs). **(A)** Representative histogram overlays showing G-CSF-R expression on splenic PMNs, macrophages (Mϕ), conventional DCs (cDCs), and CD4^+^, CD8α^+^, and DN DC subsets from B6 (plain) and B6.*Sle2c2* (dashed) mice at steady state with the control shown in gray. **(B)** G-CSF-R expression (delta geometric mean) on myeloid cell subsets. Percentage of G-CSFRhi cells among PMNs **(C)**, CD4^+^, CD8α^+^, and DN DCs **(D)**, as well as CD4^+^ T cells **(E)** from B6 (open square) and B6.*Sle2c2* (black-squares) mice at days 0 and 21 after chronic graft-versus-host disease (cGVHD) induction. **(F)** Representative FACS dot plots showing G-CSF-Rhi population within splenic PMNs, DN DCs and CD4^+^ T cells at day 21 post-cGVHD induction (*N* > 3 per subset and strain, **p < 0.01, ***p < 0.001).

We compared the expression of G-CSF-R on myeloid subsets before (Figure 3) and upon cGVHD induction (Figure S1 in Supplementary Material) between the two strains. Since a monoclonal antibody against mouse G-CSF-R has not yet been developed, we optimized a polyclonal anti-G-CSF-R antibody for flow cytometry using an anti-goat secondary antibody conjugated to AF488 (Figure S1A in Supplementary Material). The secondary antibody alone was used as control. Expression of G-CSFR was presented as Delta geometric mean obtained by subtracting the background (secondary antibody alone). At steady state, G-CSF-R expression was significantly lower on PMNs and CD8α^+^ DCs from B6.*Sle2c2* mice (Figures 3A,B). A similar trend was observed for CD4^+^ and DN DCs. G-CSF-R expression was similar in MF and total cDC between the two strains. At day 21 post-cGVHD, G-CSFR expression is increased as compared to steady state on all the above-mentioned myeloid cells, with a reduced expression only on *Sle2c2* CD4^+^ DCs (Figures S1B,C in Supplementary Material). Hence, in addition to being functionally defective (17), these results suggest that G-CSF-R is expressed at a lower level on *Sle2c2* PMN and CD8α^+^ DCs at steady state. Our G-CSFR stain allowed the discovery of a distinct G-CSFRhi population within PMNs, the percentage of which did not change after cGVHD or between strains (Figures 3C,F). A G-CSFRhi population was also found in DCs and CD4^+^ T cells. However, the frequency of this population was only greatly increased in DN DCs from B6.*Sle2c2* mice and CD4^+^ T cells from B6 mice upon cGVHD induction (Figures 3D–F), highlighting again differences in G-CSFR expression between strains.

### PMN Depletion Partially Rescued cGVHD in B6.*Sle2c2* Mice

**Figure 4:**
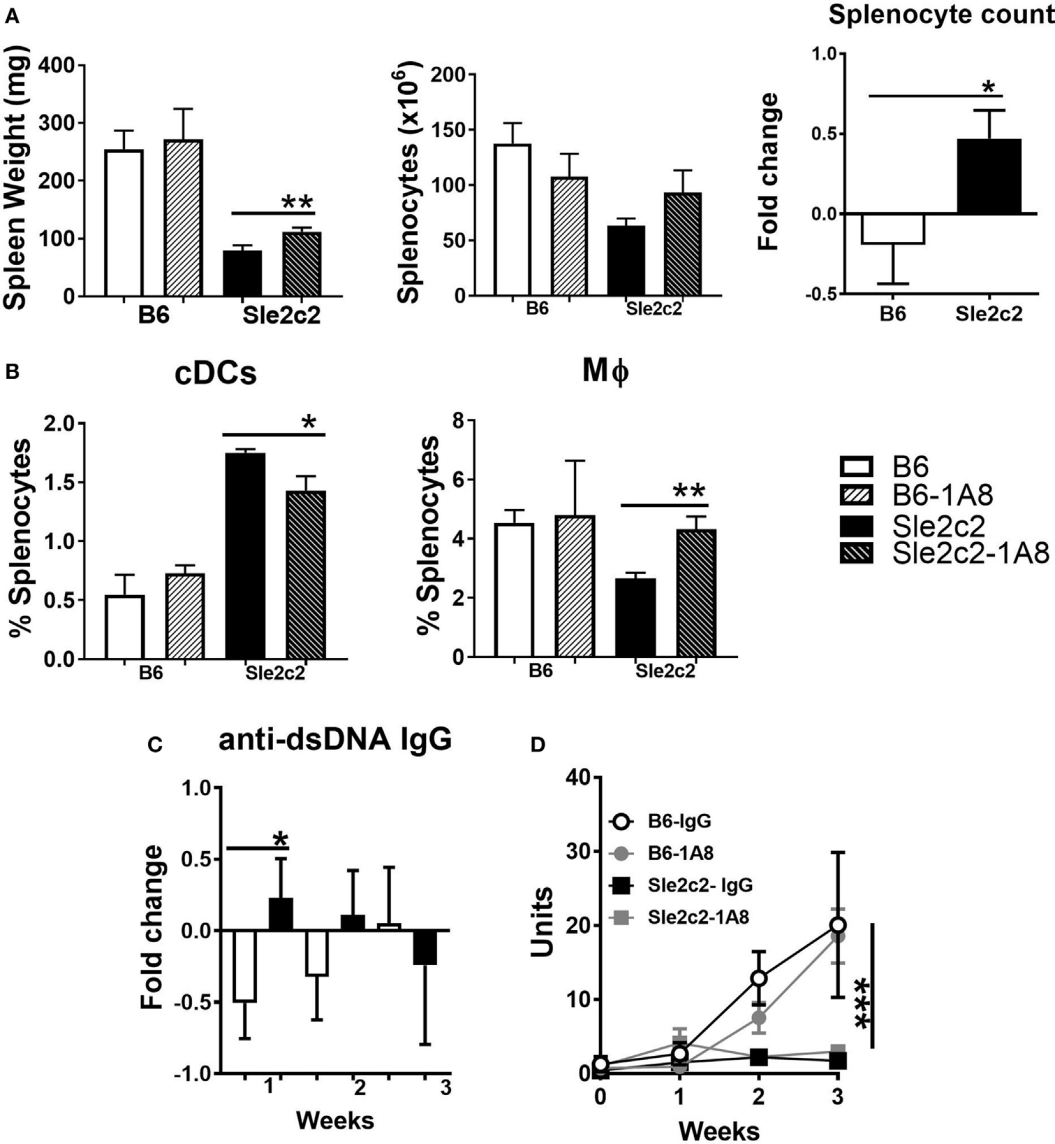
Polymorphonuclear neutrophil (PMN) depletion partially rescued chronic graft-versus-host disease (cGVHD) in B6.*Sle2c2* mice. **(A)** Spleen weight and splenocyte numbers 3 weeks after cGVHD induction in B6 and B6.*Sle2c2* mice with and without PMN depletion with the 1A8 antibody. The graph of the right show relative between mice treated with 1A8 and controls for each strain. **(B)** Frequency and numbers of conventional DCs (cDCs) and Mϕ at week 3. Fold changes were calculated as [(depleted − controls)/controls] values. *p < 0.05 indicates significant differences versus the B6 value. Serum anti-dsDNA IgG fold changes at week 1 **(C)** and time course analysis **(D)** in the four groups (*N* = 3 per treatment per strain) ***p < 0.001 two-way ANOVA between strains either treated or untreated with 1A8.

To compare the potential role for PMNs in cGVHD and assess their role in the cGVHD resistance in B6.*Sle2c2* mice, we depleted PMNs throughout the 3-week duration of the experiment. PMN depletion in B6.*Sle2c2* mice modestly increased the relative spleen weight and splenocyte numbers (Figure 4A), and the anti-dsDNA IgG response (Figure 4C), all of which are markers of cGVHD induction (43). Conversely, the number of splenocytes and the anti-dsDNA IgG levels were lowered by PMN depletion in B6 mice (Figures 4A,D), suggesting that PMNs promote cGVHD in B6 but suppress it in B6.*Sle2c2* mice. The loss of PMNs inversely affected the proportion of DCs and Mϕ in the spleen (Figure 4B). The compensatory effect observed on both Mϕ and DC frequency during PMN loss suggest a coordinated network of action between all myeloid cells during cGVHD. However, these changes were small, and B6 mice still produced a significantly stronger cGVHD response than B6.*Sle2c2* mice, with or without PMNs, as shown by serum dsDNA IgG (Figure 4D). Moreover, the loss of PMNs did not significantly alter lymphocyte activation in either strain (data not shown). Overall, these results imply that cells other than PMNs are involved in the suppression of cGVHD in B6.*Sle2c2* mice.

### B6.*Sle2c2* DCs Are More Tolerogenic Than B6 DCs

**Figure 5:**
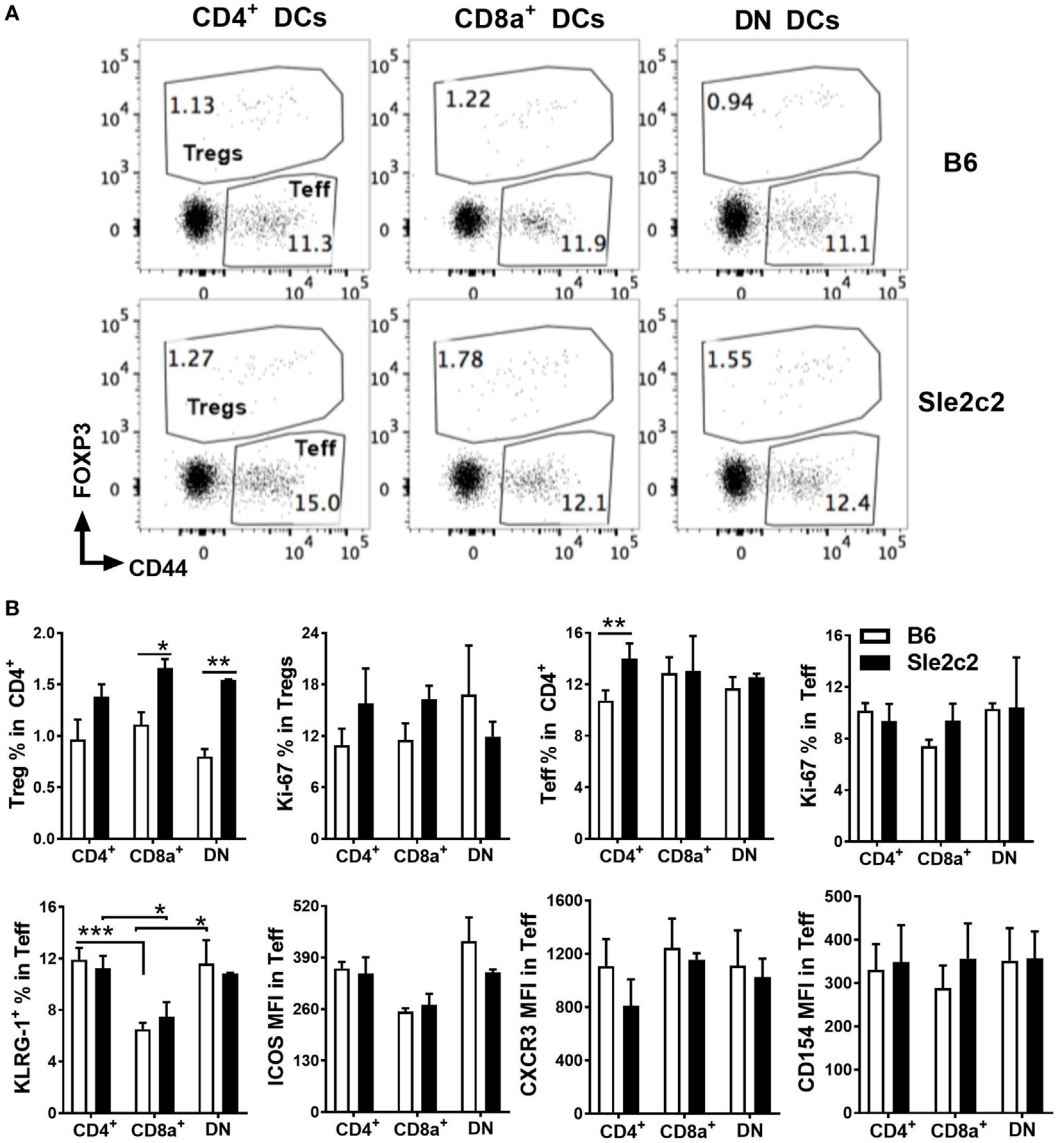
B6.*Sle2c2* dendritic cells (DCs) are more tolerogenic in antigen-specific CD4^+^ T cell cocultures. chronic graft-versus-host disease-induced B6 and B6.*Sle2c2* mice were immunized with NP-OVA on d 20. One-day later, splenic CD4^+^, CD8α^+^, and DN DCs were sorted from each strain. Each DC subset was cocultured individually with CD4^+^ OT-II cells for 72 h. **(A)** Representative FACS plots showing CD4^+^CD44^+^FOXP3^−^ effector T cells (Teff) and CD4^+^FOXP3^+^ regulatory T cells (Tregs) 72 h post coculture. **(B)** Top row: frequencies of Tregs and Teffs and percentages of Ki-67^+^ proliferating cells in each compartment. Bottom row: frequencies of KLRG-1^+^ cells and expression of ICOS, CXCR3, CD154 in the Teff population (*N* = 2–5 per subset and strain, *p < 0.05, **p < 0.01, ***p < 0.001).

The partial contribution from PMNs toward cGVHD outcomes and the preferential expansion of CD8α^+^ DCs over DN DCs in B6.*Sle2c2* mice (Figure 1B) warranted comparison of DC function between the two strains. DC antigen presentation and activation potential were tested with NP-OVA immunization 20 days after induction of cGVHD in B6 and B6.*Sle2c2* mice. One day later, sorted CD4^+^, CD8α^+^, and DN DCs were cocultured with OVA-specific OT-II CD4^+^ T cells. *Sle2c2* DCs induced higher frequencies of Foxp3^+^ regulatory T cells (Treg) than B6 DCs, with a significant difference obtained with DN and CD8α^+^ DCs (Figures 5A,B). Proliferation of Treg cells, as assessed by expression of Ki67, was similar for all DC subsets. Effector T cell (Teff) frequencies and proliferation were also similar among DC subsets and strains excepting for slightly higher Teff frequencies obtained with B6.*Sle2c2* CD4^+^ DCs (Figures 5A,B). Interestingly, CD8α^+^ DCs from both strains induced significantly lower activation (cytotoxic potential reflected by KLRG1 expression) and ICOS expression within Teff cells compared to other DC subsets while expression levels of CXCR3 and CD154 were similar among DC subsets and strains (Figure 5B). These data indicate that cGVHD resistance in B6.*Sle2c2* mice is favored by general increase in Treg-inducing capacity of spleen DC subsets and poor CD4^+^ Teff-activating potential of CD8α^+^ DCs that are increased in proportion within DC subsets of these mice as previously mentioned.

### B6.*Sle2c2* DCs Are Less Inflammatory Than B6 DCs

**Figure 6:**
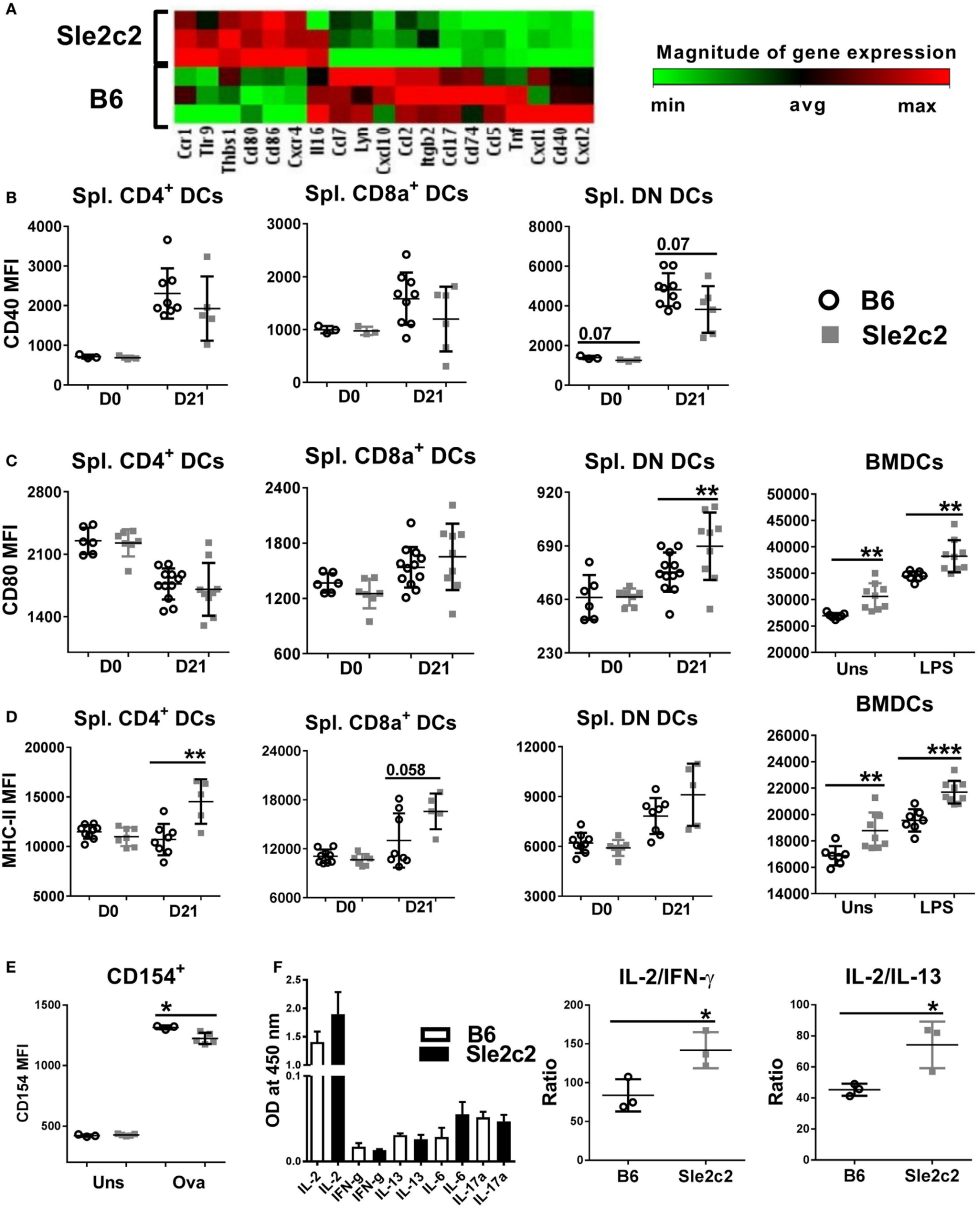
B6.*Sle2c2* bone marrow-derived DCs (BMDCs) display a less inflammatory gene expression signature than B6 BMDCs. **(A)** Differential gene expression of B6 and B6.*Sle2c2* BMDCs stimulated with 1 µg/ml LPS for 24 h (*N* = 3). CD40 **(B)**, CD80 **(C)**, and MHC-II **(D)** protein expression on unstimulated and LPS-stimulated BMDCs and the three splenic dendritic cell (DC) subsets from B6 and B6.*Sle2c2* mice before and after chronic graft-versus-host disease induction. **(E)** CD154 expression on OT-II cells cocultured with BMDCs with or without OVA peptide for 72 h. **(F)** Cytokine levels in the supernatants of the cocultures, assessed by multi-analyte ELISA. The two graphs on the right show the relative ratios of IL-2 to IFNγ and IL-13 (*N* = 3–8, *p < 0.05; **p < 0.01).

To determine the mechanisms underlying the functional differences observed between B6 and B6.*Sle2c2* DCs, we compared the transcriptional profile of LPS-stimulated BMDCs. B6.*Sle2c2* DCs expressed lower levels of genes associated with DC activation and maturation, including chemokines, such as CCL2, CCL5, and CCL7, as well as TNFα, a pro-inflammatory cytokine (Figure 6A). B6.*Sle2c2* BMDCs expressed increased levels of *Tlr9*, which has been associated with inhibitory functions (46). CD40, a co-stimulatory marker involved in T cell activation, was transcriptionally decreased in B6.*Sle2c2* BMDCs (Figure 6A), and there was a trend for reduction at the protein level both prior to and upon induction of cGVHD in B6.*Sle2c2* DN DCs (Figure 6B). The T cell co-stimulatory ligands CD80 and CD86 were increased in B6.*Sle2c2* BMDCs, which we confirmed at the protein level in both unstimulated and LPS-stimulated BMDCs and also on cGVHD-induced B6.*Sle2c2* DN DCs (Figure 6C). MHC-II expression was also higher on *Sle2c2* BMDCs, whether stimulated or not with LPS, and this was true for the three DC subsets in the spleen of B6.*Sle2c2* mice before or after cGVHD induction (Figure 6D). MHC-II and CD80 levels were even lower on splenic DC subsets from B6.*Batf3*^−/−^ mice compared to B6 controls (compare to Figure 8D), showing that CD80 and MHC-II in this cGVHD context are inversely correlated to the inflammation level. Furthermore, *Sle2c2* BMDCs induced lower levels of CD154 expression on OT-II CD4^+^ T cells cocultured in the presence of OVA peptide (Figure 6E), as well as relatively higher secretion of the anti-inflammatory cytokine IL-2 over pro- inflammatory IFN-γ and IL-13 by these T cells (Figure 6F). Overall, these results suggest that *Sle2c2* DCs exhibit a tolerogenic phenotype.

### Exogenous G-CSF Induces an Inflammatory Phenotype in B6.*Sle2c2* DC

**Figure 7:**
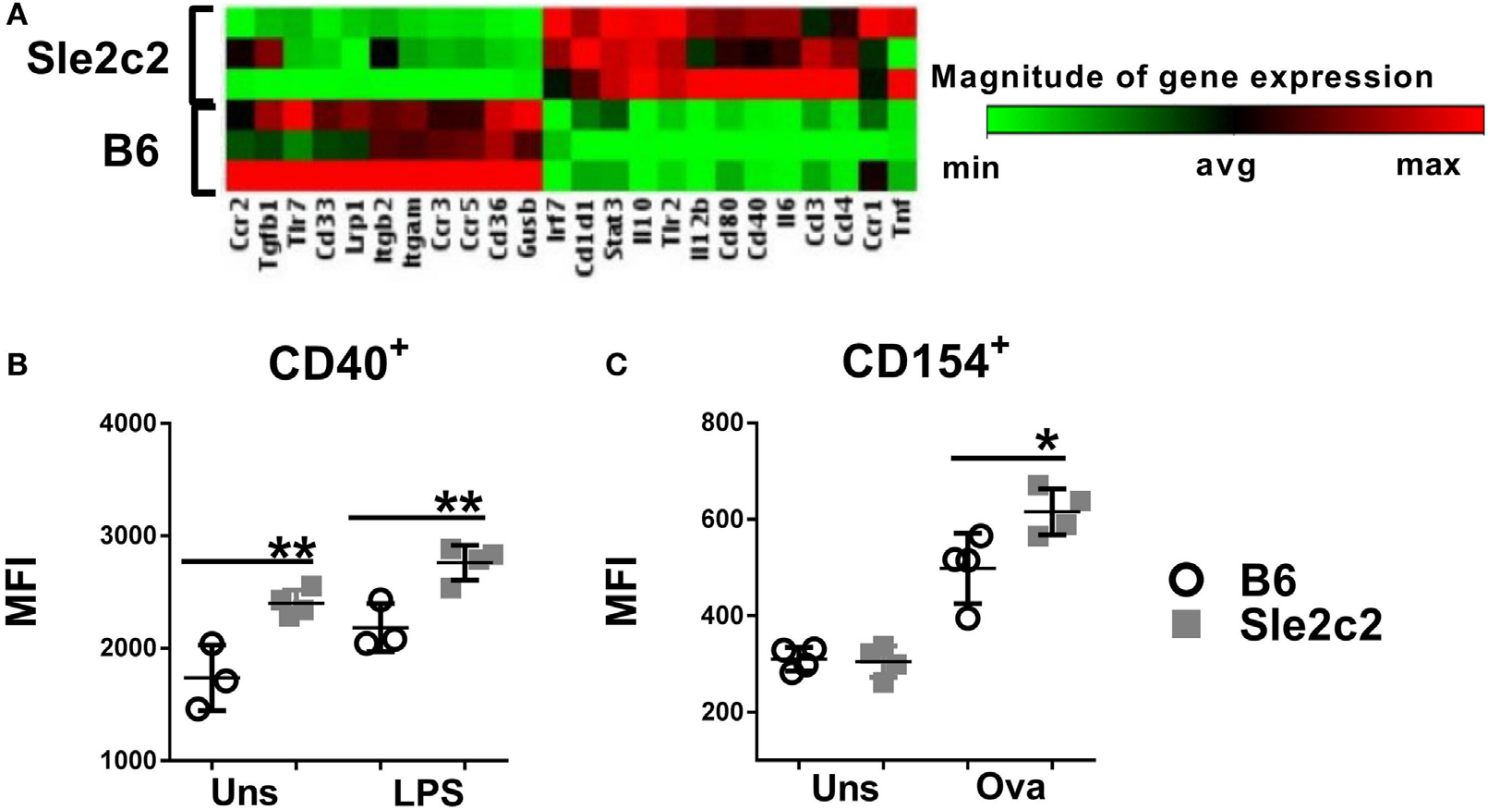
Exogenous G-CSF enhanced B6.*Sle2c2* dendritic cells functions. **(A)** Differential gene expression in bone marrow-derived DCs (BMDCs) differentiated from B6 and B6.*Sle2c2* mice treated with G-CSF (*N* = 3). **(B)** CD40 protein expression in unstimulated and LPS-stimulated BMDCs from G-CSF-treated B6 and B6.*Sle2c2* mice. **(C)** CD154 protein expression on OT-II T cells cocultured with BMDCs from G-CSF treted B6 and B6.*Sle2c2* mice, with or without OVA peptide for 72 h (*N* = 4 per group for **(B,C)**, *p < 0.05, **p < 0.01).

To avoid supra-physiological *in vitro* exposure, we differentiated BMDCs from mice treated with G-CSF 4 days earlier. BMDCs from G-CSF-treated B6.*Sle2c2* mice overexpressed several pro-inflammatory genes, such as *Tnf, Il6, Stat3, Il12b*, and *Cd40* as compared to BMDCs from G-CSF treated B6 mice (Figure 7A). The upregulation of CD40 was validated at the protein level and was maintained after exposure to LPS (Figure 7B). BMDCs differentiated from G-CSF treated B6*.Sle2c2* mice also induced a higher level of CD154 expression than BMDCs from B6 mice on OT-II CD4^+^ T cells cocultured in the presence of OVA (Figure 7C). Exogenous G-CSF was found to preferentially skew the *Sle2c2* DCs to an inflammatory phenotype over B6 DCs, further confirming the differential response to G-CSF by both strains.

### Deficiency in CD8α^+^ DCs Enhanced cGVHD Immune Activation

**Figure 8:**
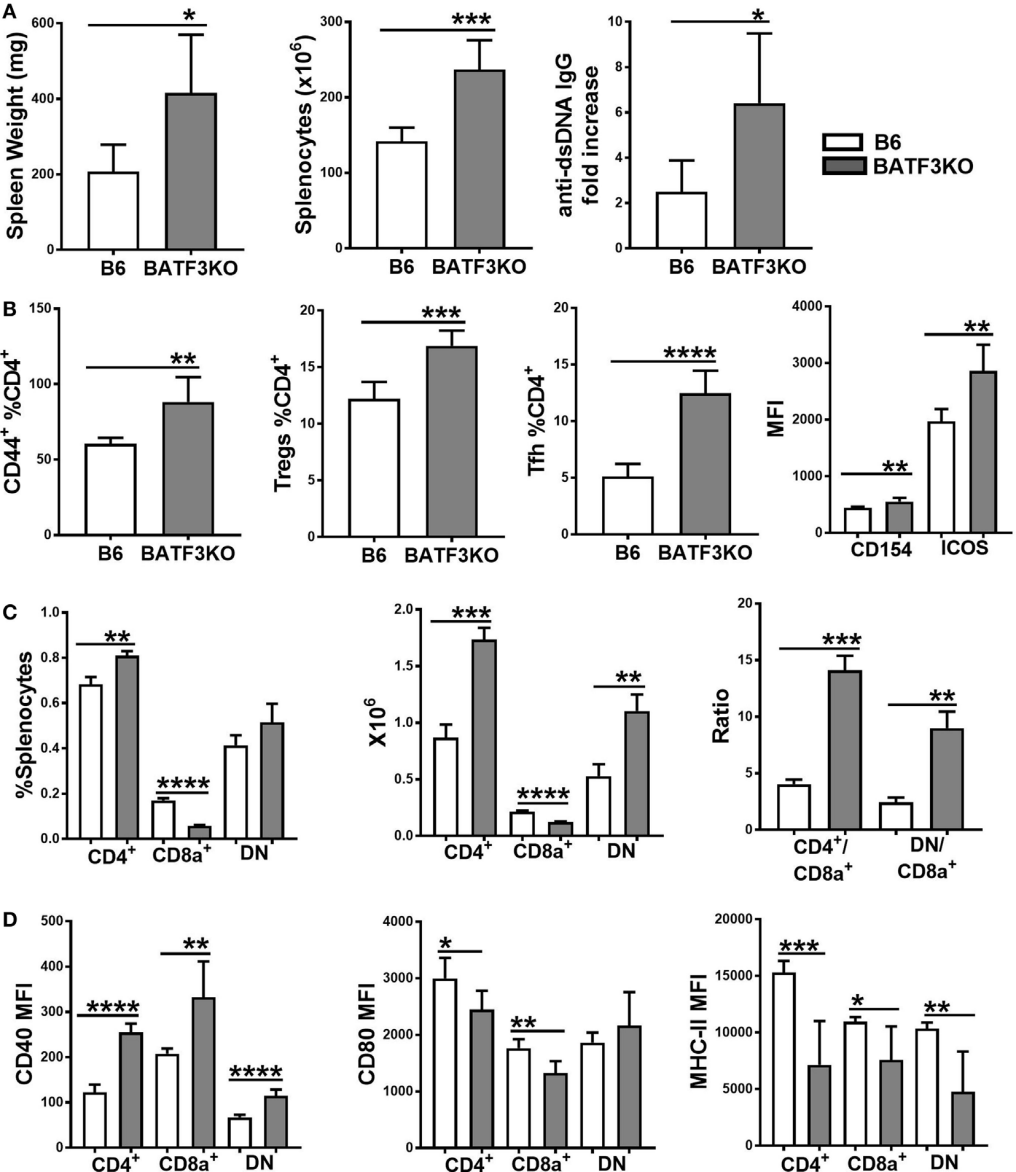
Reduction of CD8α^+^ dendritic cells (DCs) by *Batf3*-deficiency enhances bm12.cGVHD autoimmune phenotypes. Phenotypes were analyzed 21 days after chronic graft-versus-host disease (cGVHD) induction with bm12 splenocytes in B6 and B6.*Batf3*^−/−^ mice. **(A)** Spleen weight, splenocyte numbers and serum anti-dsDNA IgG fold increase from day 0. **(B)** Frequencies of effector T cell (Teff), regulatory T cells (Treg), and Tfh cells and MFI of CD154 and ICOS on Teff cells. **(C)** Frequencies, absolute numbers, and ratios of the three DC subsets. **(D)** CD40, CD80, and MHC-II expression on splenic DC subsets (*N* = 6, *p < 0.05; **p < 0.01, ***p < 0.001, ****p < 0.0001).

The preferential skewing of CD4^+^ T cells toward Treg cells in coculture with *Sle2c2* CD8α^+^ DCs and the relative expansion of this subset during suppression of cGVHD support a tolerogenic phenotype for the *Sle2c2* CD8α^+^ DCs. To test the hypothesis that CD8α^+^ DCs were involved in cGVHD suppression in B6.*Sle2c2* mice, we compared cGVHD induction between B6. *Batf3*^−/−^ mice and B6 controls. Deletion of the transcription factor *Batf3* ablates the development of CD8α^+^ DCs (47). B6. *Batf3*^−/−^ mice responded with an enhanced severity of all the hallmark phenotypes of cGVHD, including spleen weight, splenocyte numbers, and serum anti-dsDNA IgG levels (Figure 8A). CD4^+^ T cell activation was enhanced, as shown by the increased frequency of CD44^+^, Teff, and follicular helper T cells (Tfh), as well as the expression levels of ICOS and CD154 (Figure 8B). The frequency of Treg cells was also increased (Figure 8B), possibly as a consequence of the ongoing immune activation. As expected, B6.*Batf3*^−/−^ mice showed an expanded population of CD4^+^ and DN DCs relative to CD8α^+^ DCs (Figure 8C). The enhanced T cell activation was accompanied by heightened CD40 activation on all DC subsets (Figure 8D), confirming the essential role of CD40 in cGVHD (48, 49). Intriguingly, CD80 and MHC-II levels were reduced on the three DC subsets from B6.*Batf3*^−/−^ mice (Figure 8D). Overall, the genetic reduction of CD8α^+^ DCs enhanced cGVHD phenotypes, suggesting that they suppress cGVHD and could be important contributors to disease suppression in B6.*Sle2c2* mice.

### CD8α^+^ DCs Are Sufficient to Provide Resistance to cGVHD

**Figure 9:**
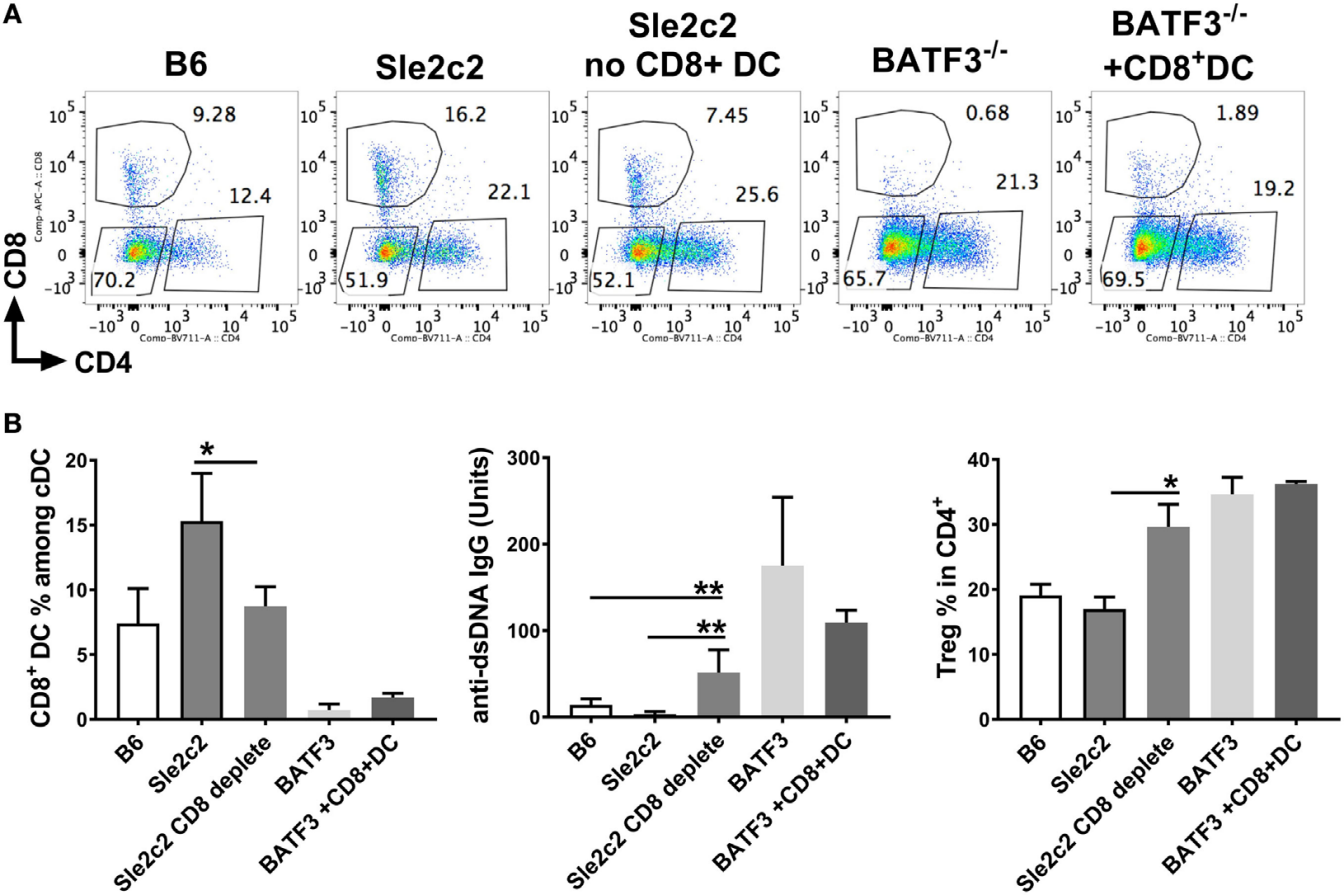
CD8α^+^ dendritic cells (DCs) are sufficient to induce resistance against chronic graft-versus-host disease (cGVHD). cGVHD was induced in indicated mice for 21 days. A sub-group of B6.*Sle2c2* was depleted with anti-CD8a depleting antibody (300 µg/mouse/i.p.) every 3 days and until day 17 while a sub-group of B6.*Batf3*^−/−^ mice was i.v. transferred with 50–70 × 10^3^ CD8α^+^ DC on days 2, 7, and 14 post-cGVHD induction. **(A)** Representative FACS plots of spleen DC subsets within each group. **(B)** Frequencies of CD8α^+^ DC among spleen DCs, anti-dsDNA IgG titers, and regulatory T cells (Treg) in CD4^+^ Tcells at day 21 post-cGVHD induction (*N* = 2–9 mice/group, *p < 0.05; **p < 0.01).

To prove a direct role of CD8α^+^ DCs in suppressing cGVHD in B6.*Sle2c2* mice, we depleted this DC subset using a depleting anti-CD8α antibody. This depletion allowed the elimination of up to 50% of splenic CD8α^+^ DCs and led to a drastic increase in anti-dsDNA IgG levels that was even greater than in the B6 controls (Figures 9A,B). This was a direct result of total splenic Tfh number increase compared to non-depleted B6.*Sle2c2* mice (Figure S2B in Supplementary Material). On the other hand, while B6. *Batf3*^−/−^ mice developed an exacerbated anti-dsDNA IgG response as expected compared to WT B6 controls, B6. *Batf3*^−/−^-mice adoptively transferred with CD8α^+^ DCs showed a reduction in anti-dsDNA IgG levels. This reduction was not significant and we predict that it could be due to the insufficient numbers of transferred CD8α^+^ DCs in these mice (Figures 9A,B). Similar to cGVHD-induced B6.*Batf3*^−/−^ mice, CD8α^+^ DC-depleted B6.*Sle2c2* mice also presented with increased frequency of Treg cells (Figure 9B), again possibly as a consequence of the ongoing immune activation. We confirmed this hypothesis by looking at the MFI levels of FOXP3 in this subset, which is directly associated with the suppressive ability of Treg cells (48). Accordingly, CD8α^+^ DC-depleted B6.*Sle2c2* mice presented with lower levels of FOXP3 in comparison to their control group (Figure S2B in Supplementary Material). Similarly, T follicular regulatory cells, which directly impact the function of Tfh cells (49, 50), were reduced in the CD8α^+^ DC-depleted *Sle2c2* group (Figure S2A in Supplementary Material), further confirming the important tolerogenic role of CD8α^+^ DCs in the context of cGVHD.

## Discussion

We have previously shown using adoptive cell transfers that the resistance to cGVHD autoimmunity in B6.*Sle2c2* mice was mediated by myeloid cells (15). Since a coding polymorphism in the gene encoding G-CSF-R is the top candidate for the genetic underpinning of this suppression (15, 17), we hypothesized that an altered G-CSF pathway in myeloid cells expressing the NZB allele of G-CSF-R was responsible for the suppression in the cGVHD-induced model of lupus. Accordingly, cGVHD suppression in B6.*Sle2c2* mice correlated with an increased prevalence of splenic CD8α^+^ DCs over the DN DCs. In addition to their altered distribution patterns, B6.*Sle2c2* DCs exhibited different phenotypes under steady state and cGVHD-induced conditions. B6.*Sle2c2* DCs expressed lower levels of G-CSF-R, exhibited immature activation patterns and preferentially skewed T cells toward a Foxp3 regulatory phenotype in antigen-specific coculture conditions. The enhanced secretion of IL-2, known for its anti-inflammatory role in SLE (51, 52), along with the decreased activation of CD154, a key target in lupus pathogenesis (53–56), could be some of the mechanisms employed by B6.*Sle2c2* DCs to curtail T cell responses in cGVHD. The difference in CD4^+^ T cell activation from cocultures performed with CD8α^+^ and DN DCs suggests that these DC subsets may participate in the suppression of T cell responses in cGVHD.

A tolerogenic phenotype has been reported for CD8α^+^ DCs, which promote peripheral tolerance either through apoptosis of self-reactive T cells (57, 58), polarization into Treg cells (59), or negative regulation of T cell proliferation and survival through the IDO-IFNγ axis (60). Confirming this predicted tolerogenic phenotype in our model, CD8α^+^ DC deficiency in B6.*Batf3*^−/−^ mice significantly enhanced the severity of cGVHD, including splenomegaly and lymphocyte activation, thus confirming the predicted tolerogenic phenotype in our model. This enhanced autoimmune activation in B6.*Batf3*^−/−^ mice corresponded with upregulation of CD40 and downregulation of all other co-stimulatory markers such as MHC-II, CD80, and CD86 in DCs, suggesting a crucial role for CD40 in the cGVHD response. Interestingly, in our model, MHC-II expression on DCs (as well as that of CD80) was always reduced in mice with a higher inflammation rate (B6.*Batf3*^−/−^ < WT B6 < B6.*Sle2c2*). However, the investigation of the involvement of CD8α^+^ DCs is still in its nascent stage, and breeding the *Batf3*^−/−^ mutation on the B6.*Sle2c2* mice with mice will provide direct evidence of the tolerogenic role for this DC subset in cGVHD suppression. Depletion of this splenic DC subset in B6.*Sle2c2* mice and adoptively transferring this DC subset to B6.*Batf3*^−/−^ mice demonstrated a direct role of CD8α^+^ DCs in providing cGVHD resistance to B6.*Sle2c2* mice. It is important to note that there is a direct correlation between presence of CD8α^+^ DCs and CD8^+^ T cell frequencies since this DC subset is essential for maintaining and activating CD8^+^ T cells (41). Accordingly, increased frequencies of spleen CD8^+^ T cells were observed in *Batf3*^−/−^ mice adoptively transferred with CD8α^+^ DCs while CD8 depletion in B6.*Sle2c2* mice eliminated both corresponding DC and T cell subsets akin to *Batf3*^−/−^ mice where CD8^+^ T cell frequencies are greatly reduced as a result of CD8α^+^ DC deficiency (data not shown). While we cannot rule out a role of CD8^+^ T cells in inducing cGVHD resistance by eliminating damaged or apoptotic cells or donor pathogenic CD4^+^ T cells, these results show that methods enhancing the proportion of this DC subset in mice might be of great use in reducing cGVHD and potentially SLE. Moreover, *Sle2c2* DCs exhibited a more tolerogenic phenotype overall as evidenced by their reduced expression of pro-inflammatory cytokine and chemokine genes. The addition of exogenous huG-CSF induced differential effects on B6 and *Sle2c2* DCs. Indeed, G-CSF treatment did not change the expression of co-stimulatory markers in B6 DCs, but it augmented the activation, antigen presentation and gene expression of pro-inflammatory markers such as Tnf, Il6, Stat3, Il12b, and co-stimulatory marker Cd40 of B6.*Sle2c2* DCs. These data suggest that exogenous G-CSF overcomes the deficiency posed by the *Csf3r* polymorphism (15, 17) to endogenous G-CSF-R pathway.

Maintaining neutrophil homeostasis is a key function of the G-CSF pathway, and both neutrophils and their progenitors bear the highest density of G-CSF-R on their surface (18, 61). Due to their ability to interact with lymphocytes and DCs, neutrophils have also been implicated in lupus pathogenesis with both pro-inflammatory (62–66) and protective roles (67–70). These observations suggested that neutrophils could be the cell type responsible for cGVHD resistance in B6.*Sle2c2* mice. However, the loss of neutrophils did not drastically alter cGVHD outcome in either B6 or B6.*Sle2c2* mice, implying that that neutrophils play a minor role in cGVHD and that cells other than neutrophils were involved in mediating the suppression to cGVHD in B6.*Sle2c2* mice. Our studies reported herein have been instrumental in better understanding the G-CSF pathway in lupus pathogenesis. Attempts to translate our knowledge of the pro-inflammatory role for G-CSF in lupus (17, 30–32) have suffered from lack of information regarding the effector cells and their cellular mechanisms. We have shown G-CSF to exacerbate cGVHD through its collective effect on multiple myeloid cell subsets, with DCs playing a primary role. With regard to the cellular mechanisms, we have shown that exogenous G-CSF reversed the tolerogenic phenotype offered by DCs expressing a polymorphic G-CSF-R allele. Furthermore, we showed that negative regulation of the G-CSF-R pathway in myeloid cells led to suppression of cGVHD. Although the polymorphism associated with the negative regulation of G-CSF-R is not conserved between mice and humans (15), the identification of effector cells in the G-CSF pathway that modulate autoimmune activation has opened new avenues for targets to suppress lupus. Blocking the G-CSF-R pathway or enhancing negative regulators of G-CSF-R pathway, such as p38 MAPK, ERK, and SOCS3, in lupus-prone mouse models could serve as a proof of concept to validate the involvement of the G-CSF pathway in lupus pathogenesis.

## Ethics Statement

This study was carried out in strict accordance with the recommendations in the Guide for the Care and Use of Laboratory Animals of the Animal Welfare Act and the National Institutes of Health guidelines for the care and use of animals in the biomedical research. All animal protocols were approved by the Institutional Animal Care and Use Committee (IACUC) of the University of Florida, Gainesville (OLAW Assurance # A3377-01).

## Author Contributions

RS and GA designed and performed experiments, and wrote the manuscript, CM and MA contributed reagents, and participated to experimental design and data interpretation, LM conceived the study, participated to experimental design, data analysis and interpretation, and wrote the manuscript.

## Conflict of Interest Statement

The authors declare that no conflicts of interest exist pertaining to the contents of this work.

## Supplementary Material

Figure S1 G-CSFR expression on innate and spleen dendritic cell (DC) subsets after chronic graft-versus-host disease (cGVHD) induction. **(A)** Representative histogram overlays showing G-CSF-R expression on Ly6G^+^CD11b^+^ polymorphonuclear neutrophils (PMNs) from a naïve B6 mouse using both primary goat anti-mouse G-CSFR and secondary AF488-conjugated anti-goat antibody (plain line), secondary anti-goat alone (gray-filled line), or without primary or secondary antibodies (dashed line). **(B)** Representative histogram overlays showing G-CSF-R expression on splenic conventional DCs (cDCs), Mϕ, PMNs and CD4^+^, CD8α^+^ and DN DC subsets in B6 (plain) and B6.*Sle2c2* (dashed) mice with the control (secondary alone) shown in gray, 21 days after cGVHD induction. **(C)** G-CSF-R expression on above-mentioned myeloid cell subsets represented as delta geometric mean (primary + secondary antibody value − secondary antibody alone value). (*N* > 3 per subset and strain, **p* < 0.05).

Figure S2 Loss of CD8α^+^ dendritic cells (DCs) breaks the resistance against chronic graft-versus-host disease (cGVHD). cGVHD was induced in indicated mice for 21 days. **(A)** Frequencies of Tfh and T follicular regulatory (Tfr) cells, ratio of Tfh/Tfr cells. **(B)** FOXP3 expression in regulatory T cells (Treg) cells from in groups described in Figure 8 at days 21 post-cGVHD induction (*N* = 2–9 mice/group, **p* < 0.05).

## Abbreviations

SLE, systemic lupus erythematosus; B6, C57BL/6J mice; bm12, B6.C-H2bm12/Khg mice; OT-II, B6.Cg- Tg(TcraTcrb) 425Cbn/J mice; *Batf3*^−/−^, 129S-*Batf3*tm1Kmm/J mice; TC, B6.NZM-Sle1NZM2410/AegSle2NZM2410/AegSle3NZM2410/Aeg/LmoJ; BM, bone marrow; DC, dendritic cell; pDC, plasmacytoid dendritic cell; MF, macrophage; PMN, polymorphonuclear neutrophil; TN, CD44^−^CD62L^+^ naïve T cells; Treg, regulatory T cells; Teff, effector T cells; TEM, CD44^+^CD62L^−^ CD4^+^ effector memory; CD8α^−^ CD4^−^ (DN) DC.
