# Supplementary figures and images for: Protective Role of Myeloid Cells Expressing a G-CSF Receptor Polymorphism in an Induced Model of Lupus
Source: Front Immunol. 2018 May 9;9:1053. doi: 10.3389/fimmu.2018.01053 (PMC5954343; doi:10.3389/fimmu.2018.01053)

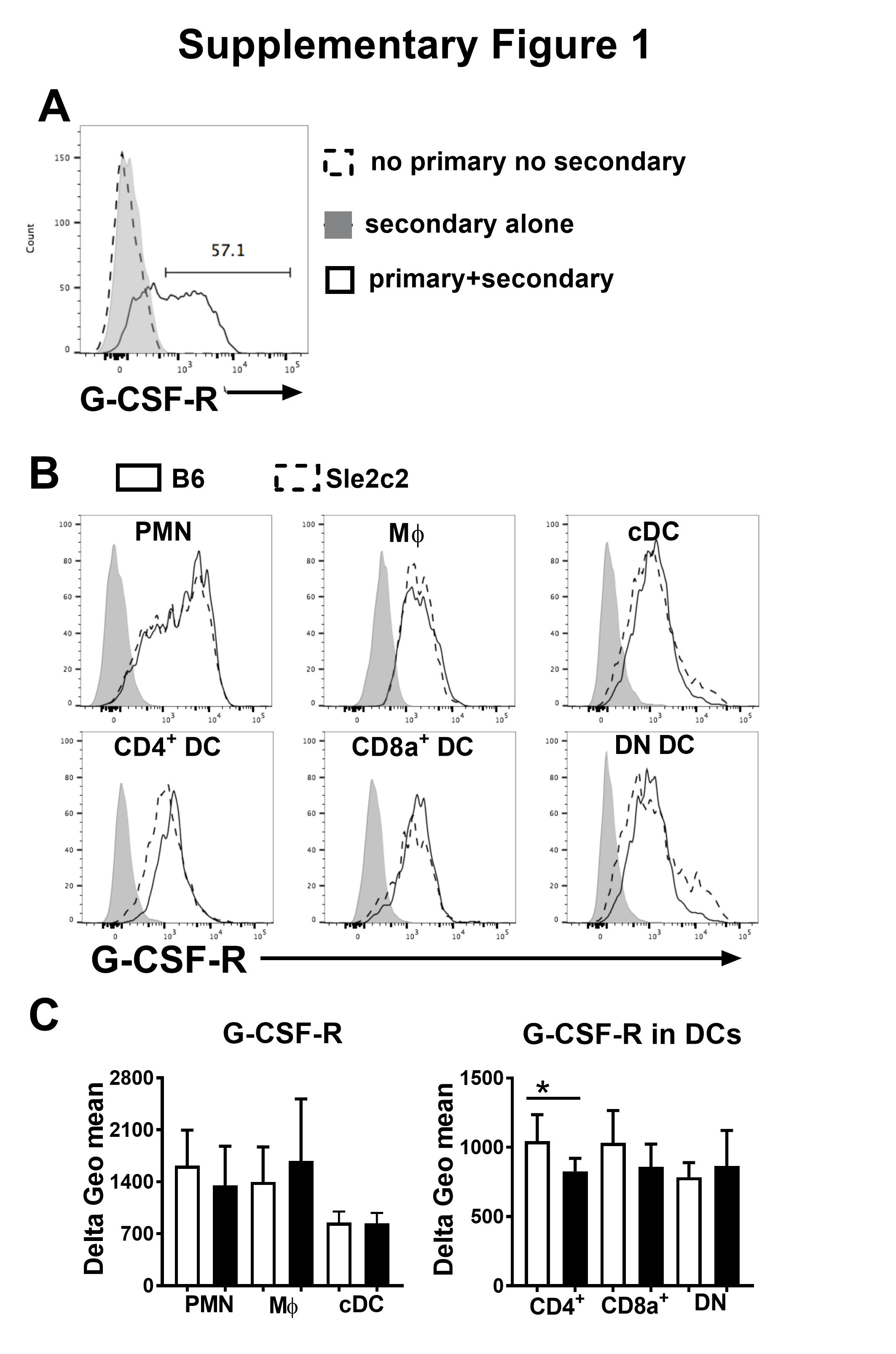

Supplement: Figure S1 — G-CSFR expression on innate and spleen dendritic cell (DC) subsets after chronic graft-versus-host disease (cGVHD) induction. (A) Representative histogram overlays showing G-CSF-R expression on Ly6G+CD11b+ polymorphonuclear neutrophils (PMNs) from a naïve B6 mouse using both primary goat anti-mouse G-CSFR and secondary AF488-conjugated anti-goat antibody (plain line), secondary anti-goat alone (gray-filled line), or without primary or secondary antibodies (dashed line). (B) Representative histogram overlays showing G-CSF-R expression on splenic conventional DCs (cDCs), Mϕ, PMNs and CD4+, CD8α+ and DN DC subsets in B6 (plain) and B6.Sle2c2 (dashed) mice with the control (secondary alone) shown in gray, 21 days after cGVHD induction. (C) G-CSF-R expression on above-mentioned myeloid cell subsets represented as delta geometric mean (primary + secondary antibody value − secondary antibody alone value). (N > 3 per subset and strain, *p < 0.05). [file Image_1.jpeg]

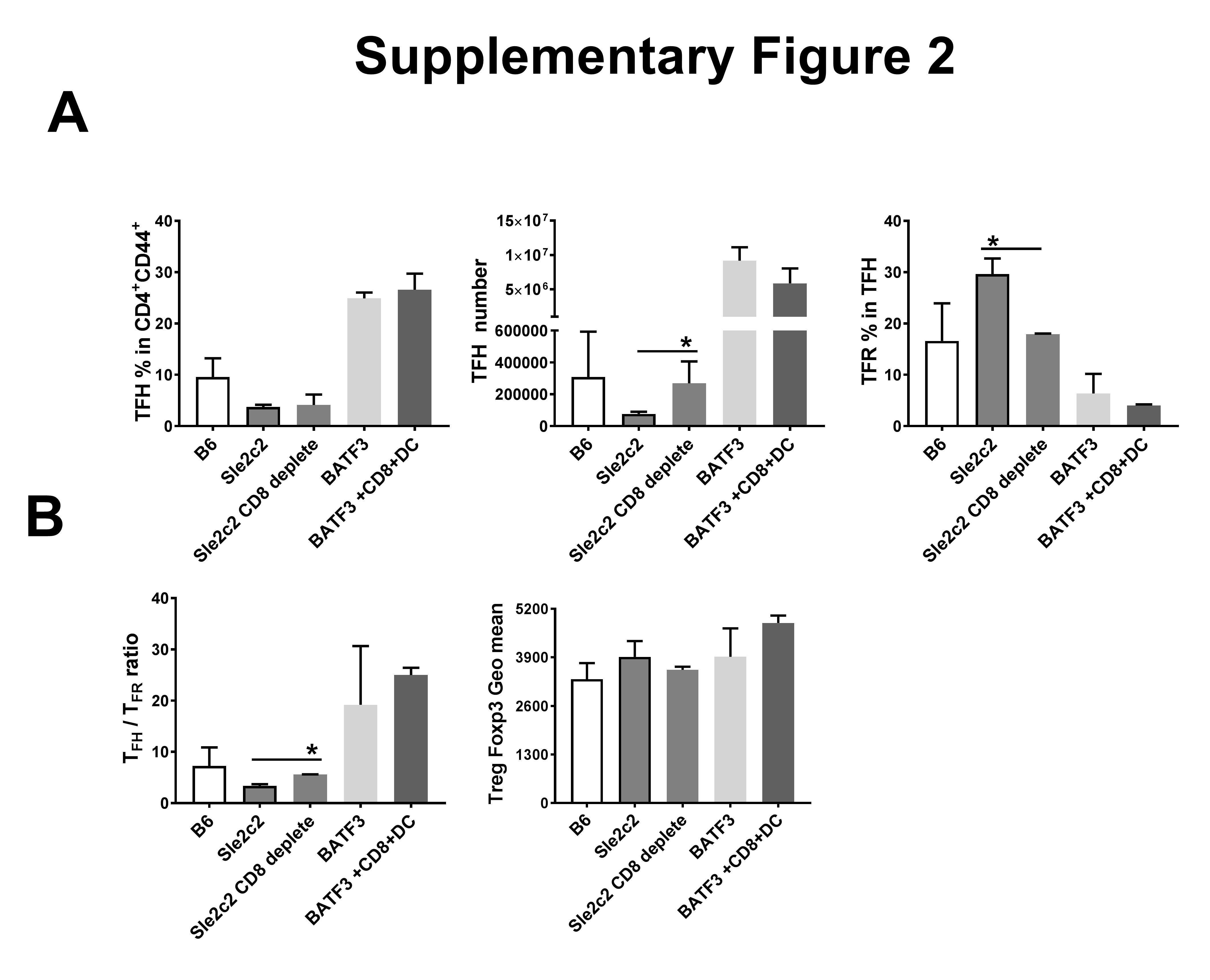

Supplement: Figure S2 — Loss of CD8α+ dendritic cells (DCs) breaks the resistance against chronic graft-versus-host disease (cGVHD). cGVHD was induced in indicated mice for 21 days. (A) Frequencies of Tfh and T follicular regulatory (Tfr) cells, ratio of Tfh/Tfr cells. (B) FOXP3 expression in regulatory T cells (Treg) cells from in groups described in Figure 8 at days 21 post-cGVHD induction (N = 2–9 mice/group, *p < 0.05). [file Image_2.jpeg]
